# Investigation of a norovirus outbreak among hospital staff in Zhejiang, China: tracing the source to contaminated “red bean cake”

**DOI:** 10.3389/fpubh.2025.1631091

**Published:** 2025-07-24

**Authors:** Xiaojian Duan, Jing Wang, Yi Wang, Zhaokai He, Xiaobin Ren, Zhe Wang, Qingjun Kao, Kai Song, Liangliang Huo

**Affiliations:** Hangzhou Center for Disease Control and Prevention (Hangzhou Health Supervision Institution) Institute of Infectious Disease Control and Prevention, Hangzhou, China

**Keywords:** norovirus, healthcare workers, case-control study, outbreak investigation, source tracing

## Abstract

While nosocomial norovirus transmission in hospitalized patients is well characterized, its transmission dynamics among HCWs remain poorly documented. This investigation of HCW-focused norovirus transmission provides critical epidemiological evidence to refine infection control protocols for gastroenteritis in healthcare settings. This study utilized a retrospective case–control design to systematically analyze outbreak transmission dynamics. Structured questionnaires were implemented ≤72 h post-symptom onset to capture dietary exposures during the three-day exposure window, minimizing recall bias. Potential high-risk dining periods and food items were further analyzed via a case-control study. The outbreak investigation identified 52 cases, including 48 HCWs and 4 cafeteria staff, yielding an overall attack rate of 2.21% (52/2352). Epidemiological evidence supports a point-source origin, as demonstrated by the single-peak epidemic curve. Case–control analysis revealed the lunch on 19th June as the primary exposure window (statistically significant OR = 25.21; 95% CI: 3.35–189.69), with the “red bean cake” served in the implicated meal being the significantly associated food item (OR = 1248.75; 95% CI: 170.64–9138.33). RT-qPCR confirmed norovirus GII RNA in clinical specimens from cases and the implicated “red bean cake” food sample. These findings definitively established the “red bean cake” as the outbreak’s etiological source.

## 1 Introduction

Human norovirus spreads primarily via the fecal-oral route, including direct person-to-person contact and ingestion of contaminated food or water (1, 2). Owing to its high transmissibility and low infectious dose (approximately 18–1,000 viral particles) (3), norovirus outbreaks frequently occur in semi-closed or closed settings (e.g., hospitals, cruise ships and childcare centers) (4). In healthcare settings, outbreaks predominantly affect in patients; infections among healthcare workers (HCWs) are less frequent. The incubation period ranges from 12 to 72 h, with a median of 36 h and mean duration of 12–48 h (5). Norovirus displays high environmental stability. Studies confirm norovirus retains infectivity for over 61 days in controlled laboratory settings and remains detectable in groundwater for more than 3 years, demonstrating exceptional environmental persistence (6). Since 2013, norovirus has emerged as the leading cause of acute gastroenteritis outbreaks in China (7). In healthcare settings, containing norovirus outbreaks poses distinct challenges. Given its high transmissibility, asymptomatic or mild infections can drive healthcare-associated spread. Currently, no antiviral therapies or vaccines are approved for norovirus prophylaxis or treatment (8). Norovirus demonstrates seasonal prevalence, with peak incidence typically occurring between October and March of the following year (9). In immunocompetent hosts, norovirus infection is generally self-limiting, presenting with symptoms such as diarrhea, vomiting, nausea, low-grade fever, and abdominal cramps. Symptoms typically resolve spontaneously within 2–4 days post-infection (10). Thus, non-pharmaceutical interventions (NPIs)—including case isolation, hand hygiene, environmental disinfection, and food safety protocols—are critical to curbing transmission and limiting environmental contamination (11).

However, infants, older adults, and immunocompromised patients are at risk of severe complications, including life-threatening outcomes (12). In high-density congregate settings such as long-term care facilities, childcare centers, and hospitals, outbreak risk escalates due to increased transmission potential. Such outbreaks impose substantial economic costs and contribute disproportionately to the public health burden (13). Containment of norovirus outbreaks in healthcare facilities is particularly challenging due to high occupant density and vulnerable populations. Ineffective outbreak management may disrupt clinical operations, incur substantial financial losses, and damage institutional reputation. During nosocomial norovirus outbreaks, prompt containment through rapid implementation of multimodal interventions are critical priorities. Delayed response (>72 h from index case identification) increases secondary attack rates by 3.2-fold (95% CI: 1.8–5.7) according to World Health Organization (WHO) outbreak management guidelines (14). Effective control measures not only mitigate nosocomial transmission risks (OR = 0.24, 95% CI: 0.12–0.46) but also preserve healthcare system functionality by reducing ward closures (median closure duration: 7 vs. 14 days in uncontrolled outbreaks, *p*-value<0.01) (15).

The containment of nosocomial norovirus outbreaks necessitates two critical priorities; rapid implementation of multimodal containment strategies and precision source attribution through molecular epidemiological methods. These priorities are vital to safeguarding patient and healthcare worker safety while maintaining hospital operational continuity. The primary objective of this study was to investigate the source of a suspected norovirus outbreak among HCWs at a tertiary hospital in Zhejiang, China. Through a comprehensive epidemiological investigation combining case-control methodology, environmental assessment, and laboratory confirmation, we aimed to identify the specific exposure source, characterize outbreak risk factors, and provide evidence-based recommendations to strengthen infection control protocols for gastroenteritis outbreaks in healthcare settings.

## 2 Materials and methods

### 2.1 Research subjects

HCWs, canteen workers, and all inpatients at a hospital in Zhejiang Province.

### 2.2 Methods

#### 2.2.1 Case definition

Suspected cases were defined according to the Technical Guidelines for Norovirus Infection Outbreak Investigation, Prevention and Control (2015) (16) as HCWs or food service staff experiencing ≥3 loose/watery stools within a 24-h period and/or ≥2 vomiting episodes during a 24-h observation window, with symptom onset occurring between 17th and 23rd June, 2024. This interval encompasses the maximum incubation period (72 h) before the first identified case (19th June) and after the last case (20th June), ensuring comprehensive case ascertainment. The peak outbreak period remained 19th--20th June. Asymptomatic individuals, even if later RT-qPCR testing positive for norovirus, were not classified as cases. Confirmed cases were laboratory-confirmed through detection of norovirus nucleic acid via Real-time fluorescent quantitative polymerase chain reaction (RT-qPCR) in stool, anal swab, or vomitus specimens.

#### 2.2.2 Case search

The cases were identified through multiple methodologies. Querying the hospital’s Healthcare Department, reviewing medical records from the gastrointestinal and fever outpatient clinics, probing potential clusters of symptomatic cases, conducting interviews with canteen personnel, and performing individual case investigations via telephone interviews. Case identification was conducted through 23rd June, 2024, extending one maximum incubation periods (72 h) beyond the last identified case onset to maximize detection of epidemiologically linked cases.

#### 2.2.3 Hygiene investigation

A comprehensive assessment was conducted through interviews with key stakeholders, including physicians from the hospital’s healthcare department, administrators, and canteen managers, supplemented by on-site inspections of the hospital’s drinking water supply system and evaluations of the canteen’s spatial layout, procurement protocols, food preparation processes, storage conditions, and sanitation practices.

#### 2.2.4 Case-control study

Individuals fulfilling the diagnostic criteria for both suspected and confirmed cases were classified into the case cohort. Controls were recruited from the same department and floor level as the cases to form the control cohort. The retrospective case-control study utilized a 1:2 matched design. Controls were matched to cases by occupation, sex, age, and workspace location. Where >2 eligible controls existed per case within matched strata, all were retained to maximize statistical power. Matching variables were selected to control for potential confounding from occupational exposure gradients, spatial proximity to the outbreak epicenter. A retrospective dietary analysis was conducted to assess meal consumption patterns and identify high-risk food exposures within the three-day period prior to symptom onset.

#### 2.2.5 Sample collection and laboratory testing

Rectal swabs and environmental swabs (cutting boards and knives used for portioning, countertops in the pastry assembly area and food handler gloves) were collected using sterile nylon-flocked swabs and immediately placed in 3 mL of viral transport medium (Virocult®, Medical Wire & Equipment). Fecal specimens were collected in sterile, leak-proof containers without preservatives. Five leftover ‘red bean cake’ samples (~ 50 g of each item) were collected aseptically into sterile Whirl-Pak® bags. All specimens were stored at 4°C within 30 min of collection and transported on triple-layer ice packs (maintaining 2–8°C) to the Hangzhou Municipal CDC laboratory within 4 h of collection. Five portions of leftover ‘red bean cake’ from the implicated batch (served on 19 June) were sampled using sterile nylon-flocked swabs. Each sample was collected by thoroughly swabbing ~20 cm^2^ of the pastry surface in a standardized S-pattern. All five swabs were placed into a single 3 mL vial of viral transport medium (Virocult®). Pathogen screening utilized multiplex RT-qPCR and culture-based biochemical assays for bacterial detection (*Bacillus cereus, Salmonella* spp.*, Shigella* spp.*, Staphylococcus aureus, Vibrio parahaemolyticus, or Campylobacter* spp.).

#### 2.2.6 Norovirus testing

Viral RNA extraction was performed using the QIAamp Viral RNA Mini Kit (Qiagen, Hilden, Germany) according to manufacturer’s protocol. Briefly, 140 μL of sample supernatant (clinical specimens or composite food swab sample was vortexed vigorously for 60 s and centrifuged at 5000 rpm for 5 min.) was lysed with AVL buffer containing carrier RNA, followed by ethanol precipitation. RNA was bound to silica membranes, washed, and eluted in 60 μL AVE buffer.

Norovirus detection employed multiplex RT-qPCR targeting conserved regions of the ORF1-ORF2 junction. Reactions used the QuantiTect Multiplex RT-PCR Kits (Qiagen, Hilden, Germany) in 25 μL volumes containing (Table 1) (16).

**Table 1:** Formulation protocol for the dual reaction system of norovirus (QuantiTect Multiplex RT-PCR Kits).

Component	Volume (μL)	Final concentration (nM)
RNase-free water	3	
Cog 1F (10 μM)	1	400
Cog 1R (10 μM)	1	400
Ring 1A (10 μM)	0.5	200
Cog 2F (10 μM)	1	400
Cog 2R (10 μM)	1	400
Ring 2 (10 μM)	0.5	200
RNase-free water	4.25	
QuantiTect Multiplex RT Mix	0.25	
2 × QuantiTect Multiplex RT-PCR Master Mix	12.5	1x

According to the instructions of the QuantiTect Multiplex RT-qPCR Kits, the RT-qPCR reaction condition was reverse transcription at 50°C for 30 min; pre-denaturation at 95°C for 15 min; denaturation at 94°C for 45 s, annealing at 60°C for 30 s, 45 cycles and negative, positive, and blank controls were established. The cyclic threshold value (Ct value) ≤ 40 and a typical “S” curve were judged as positive, indicating that norovirus nucleic acids were detected in the samples. Positive nucleic acids were stored at −80°C (Table 2).

**Table 2:** Primers and probes for multiplex fluorescent quantitative RT-qPCR amplification of norovirus.

Genotype	Primers/probes	Sequence (5′-3′)
GI	Cog 1F	CGY TGG ATG CGN TTY CAT
	Cog 1R	CTT AGA CGC CAT CAT CAT TYA C
	Ring 1A	FAM-AGA TYG CGA TCY CCT GTC CA-BHQ1
GII	Cog 2F	CARGAR BCN ATG TTY AGR TGG ATG AG
	Cog 2R	TCG ACG CCA TCT TCA TTC ACA
	Ring 2	CY5-TGG GAG GGC GAT CGC AAT CT-BHQ2

#### 2.2.7 Statistical analysis

Data collection and organization were conducted using Microsoft Excel 2019 (Microsoft Corp., Redmond, WA, USA), which was also utilized to generate the epidemic curve to visualize temporal case distribution. Statistical analyses were conducted using SPSS 26.0 (IBM Corp., Armonk, NY, USA). The case-control study employed 1:2 matching with caliper restrictions. Controls were matched to cases by occupation (exact matching), sex (exact matching), age (±5 years), and workspace location (building and floor, stratified matching). The chi-square test was applied to compare categorical variables across study groups. A two-tailed *p*-value threshold of <0.05 was established as the criterion for statistical significance.

## 3 Results

### 3.1 Basic information of the hospital

The hospital employs a total workforce of 2,352 individuals, including 622 males and 1,730 females. The hospital complex is divided into two primary structures: a comprehensive medical facility and an administrative building, positioned on opposing northern and southern sides of the central thoroughfare.

The hospital’s sole cafeteria is exclusively tasked with providing three meals daily to clinical and administrative personnel. Located on the first floor of the Administrative Building, the cafeteria is divided into distinct eastern and western dining halls. These spaces are linked through a shared first-floor lobby, offering a combined seating capacity of 550 individuals. A workforce of 103 employees operates the facility, ensuring full operational coverage during service hours. The facility is equipped with professional insect control systems, and all windows are fitted with insect-proof screens. Adequate ventilation further ensures a hygienic and comfortable dining environment for personnel. These infrastructural enhancements strengthen the hospital’s logistical support framework and support the seamless functioning of daily operations.

### 3.2 Epidemic overview

**Figure 1:**
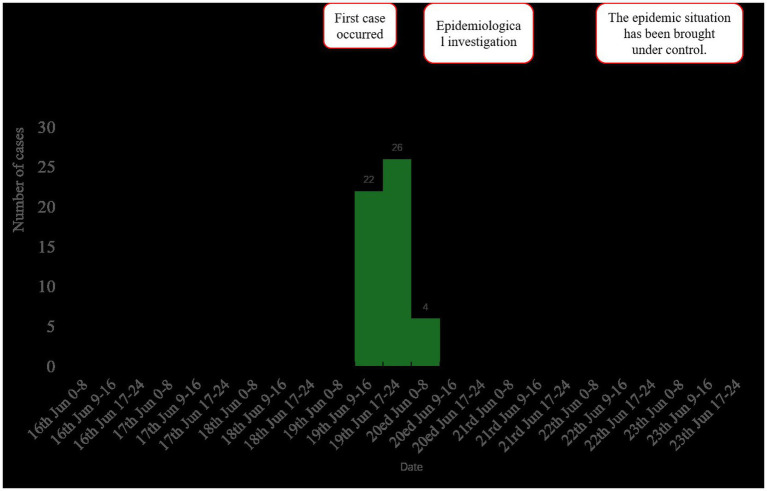
Epidemic curve of norovirus GII cases (*n* = 52) by date of symptom onset, showing peak transmission associated with cafeteria exposure on 19th June, 2024.

As of 17:00 h on 20th June, 56 hospital staff members presented with symptoms including vomiting and diarrhea. Of these, 52 met the case definition, with six cases laboratory-confirmed. The attack rate was calculated as 2.21% (52/2352). Demographically, the 52 cases included 48 HCWs and 4 cafeteria staff. Among the 52 cases, 49 sought medical care at a hospital. Among patients requiring treatment, 47 individuals underwent routine blood tests. Results demonstrated that 41 of 52 cases (78.85%; 41/52) exhibited elevated white blood cell counts, whereas the remaining 11 cases (21.15%) showed normal values. Additionally, 35 patients underwent routine fecal testing, with 17 cases (48.57%; 17/35) testing positive for occult blood and exhibiting fecal leukocytes. The remaining 18 cases (51.43%) displayed normal results. Notably, no severe complications or hospitalizations were reported. Clinical presentations were generally mild, with no significant disruptions to hospital operations or adverse health effects among personnel. The outbreak period spanned from 14:00 on 19th June to 06:00 on 20th June. No cases with onset after 20th June were identified despite active surveillance through 23rd June (Figure 1).

### 3.3 Clinical manifestations

The clinical manifestations in the 52 cases primarily included diarrhea, vomiting, and abdominal pain. Additionally, some patients exhibited symptoms such as fever and nausea (Table 3).

**Table 3:** Clinical manifestations of 52 cases in a norovirus enteritis outbreak among HCWs, Zhejiang, 2024.

Clinical manifestations	Number of cases	Rate (%)
Diarrhea	50	96.15
Vomiting	34	65.38
Abdominal pain	18	34.62
Fever	13	25
Nausea	3	5.77

### 3.4 Epidemiological characteristics

#### 3.4.1 Temporal distribution

The outbreak period was defined from the first case onset (14:00, 19th June) to the last case onset (06:00, 20th June). Active surveillance confirmed no additional cases occurred after this period. The interval between the onset of the first and last cases was 16 h. The majority of cases, specifically 46 out of 52 (88.46%), had a concentrated onset period from 12:00 to 20:00 on 19th June.

#### 3.4.2 Spatial distribution

Cases clustered across two buildings: 84.62% (44/52) in the comprehensive medical building and 15.38% (8/52) in the administrative building. The attack rate among staff in the medical building was 2.30% vs. 2.04% in the administrative building. No significant difference in attack rates between staff in the two buildings was observed (*χ*^2^ = 0.06, *p*-value = 0.80). Floors were stratified into low (1–7), middle (8–14), and high (15–21) stories. Corresponding attack rates were 2.46, 1.33, and 2.22%, respectively. No significant difference in attack rates across floor levels was identified (*χ*^2^ = 2.21, *p*-value = 0.33) (Table 4).

**Table 4:** Case characteristics in a norovirus enteritis outbreak among HCWs, Zhejiang, 2024.

Characteristics	Groups	Number of cases	Attack rate (%)	*χ* ^2^	*p*-value
Gender	Male	11	1.77	0.77	0.38
	Female	41	2.37		
Occupation	HCWs	48	2.30	0.43	0.51
	Canteen staff	4	0.76		
Building	Administration building	8	2.04	0.06	0.80
	Medical building	44	2.30		
Floor	Low	34	2.46	2.21	0.33
	Middle	7	1.33		
	High	9	2.22		

#### 3.4.3 Population distribution

The cohort included 48 HCWs (attack rate: 2.30%) and 4 canteen staff (management staff, not chefs, attack rate: 0.76%); no significant difference in attack rates by occupation was observed (*χ*^2^ = 0.43, *p*-value = 0.51). 11 male cases (attack rate: 1.77%) and 41 female cases (attack rate: 2.37%) were identified. No significant difference in attack rates by sex was detected (*χ*^2^ = 0.77, *p*-value = 0.38). Age ranged from 21 to 59 years, with a median of 29 years (Table 4).

### 3.5 Field hygienic investigation

#### 3.5.1 Water usage

Hospital staff use three daily water sources: municipal tap water, barreled water, and mineral water. Drinking water is distributed through water dispensers. The comprehensive medical building contains 23 floors, each with two water dispensers, totaling 46 dispensers. The administrative building has 11 floors, with one water dispenser per floor. Water from the dispensers is filtered using replaceable filter elements, which are replaced every 6 months. Bottled water, primarily the Nongfu Spring brand, is independently procured by individual departments. Staff occasionally purchase beverages individually.

#### 3.5.2 Canteen hygiene

**Figure 2:**
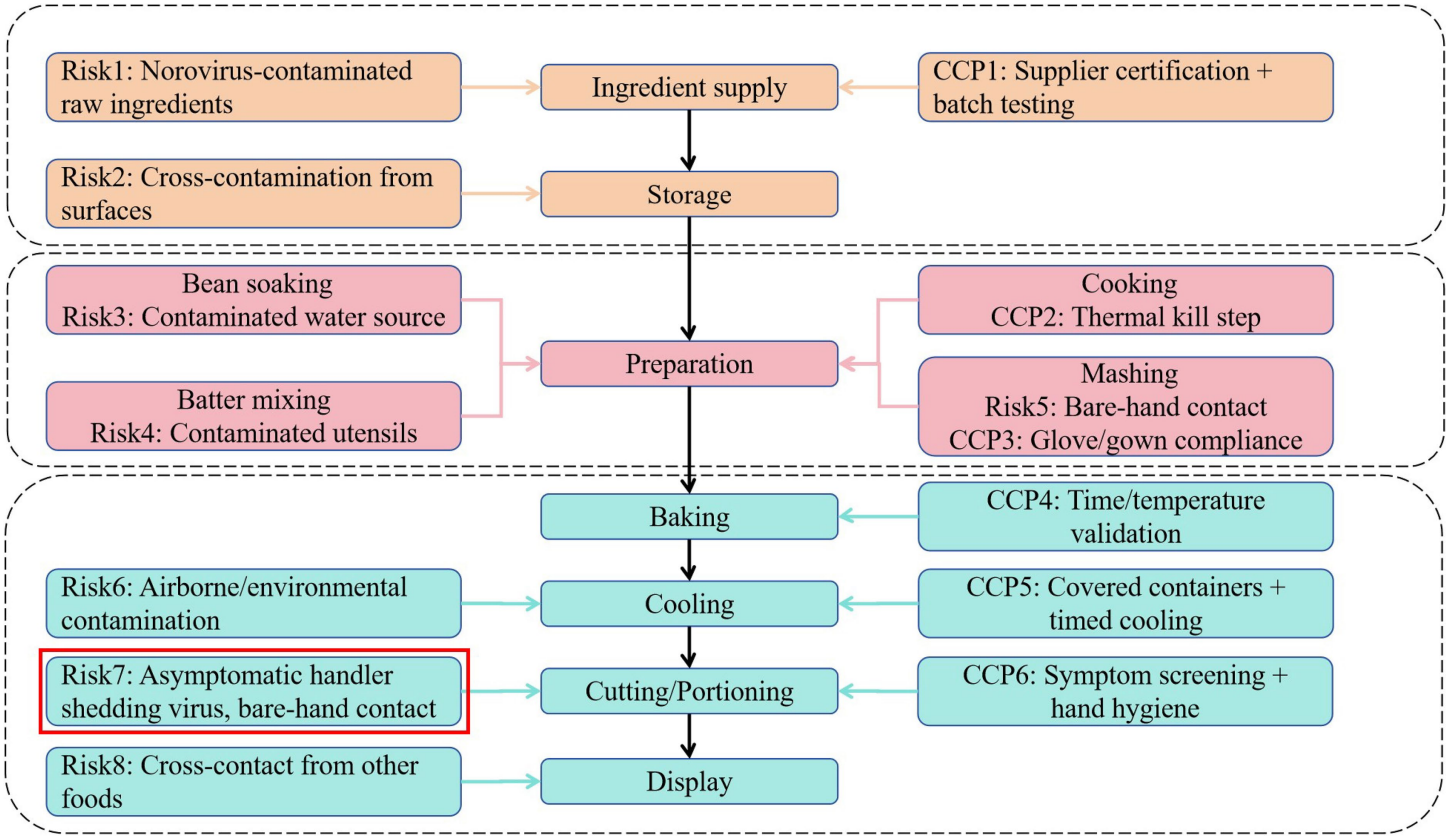
Hygienic process flow and hazard analysis for “red bean cake” preparation. CCP: critical control point, as defined in the HACCP (Hazard Analysis and Critical Control Points) system.

The cafeteria employs 103 staff. It holds a valid catering service license, and all staff maintain current health certifications. The facility features insect-proofing measures and maintains detailed logs for inventory management and disinfection protocols. Access is restricted to hospital personnel and cafeteria staff; inpatients are prohibited from using the facility. Hospital personnel stated that, apart from four cafeteria staff who presented with vomiting and diarrhea on 19th June, no additional cases of comparable symptoms were reported among cafeteria personnel. A review of the cafeteria’s menu during the 3 days preceding symptom onset showed no inclusion of high-risk foods such as raw or undercooked seafood, which are commonly associated with foodborne illness. Although food underwent adequate thermal processing, the sale of in-house prepared pastries was noted. The hygienic process flow and hazard analysis critical control points for the preparation of “red bean cake” are shown in Figure 2. Following outbreak confirmation (17:00, 20 June), terminal disinfection was implemented in three stages. Immediate containment (20 June, 18:00–22:00), all food preparation surfaces (countertops, cutting boards, utensils) were scrubbed with detergent, rinsed, then disinfected using 1,000 mg/L chlorine solution (sodium hypochlorite) applied via saturated cloths with 30-min contact time. Air and non-food contact surfaces (21 June, 08:00–12:00), ultraviolet (UV-C) irradiation for 60 min in sealed preparation areas and Floor/wall disinfection using 500 mg/L chlorine via electrostatic sprayers.

#### 3.5.3 Case-control study

Epidemiological investigation identified consumption of the cafeteria-served “red bean cake” during lunch on 19th June as common to all 6 norovirus-positive cases. This item was classified as a suspected infection source and prompted inclusion in a retrospective 1:2 matched case-control study. The study included 47 cases and 113 controls. Deviations from the planned 1:2 ratio occurred where >2 eligible controls existed within matched strata (*n* = 19 cases), enhancing power without compromising matching integrity. The study assessed meal timing and suspected food consumption during the three-day period preceding symptom onset. Analysis identified lunch on 19th June as the highest-risk exposure, yielding an odds ratio (OR) of 25.21 (95% CI: 3.35–189.69). Stratified analysis of the “red bean cake” consumed that day demonstrated an OR of 1248.75 (95% CI: 170.64–9138.33) (Table 5).

**Table 5:** Exposure analysis of meal times and food items in a norovirus outbreak among HCWs, Zhejiang, 2024.

Date	Exposure factors	Cases (*n* = 47^1^)		Control (*n* = 113^2^)		OR^3^	95% CI^4^
		Exposed population	Exposure rate (%)	Number of exposed people	Exposure rate (%)		
17th June	breakfast	9	19.15	35	30.97	0.53	0.23–1.21
	lunch	24	51.06	83	73.45	0.38	0.19–0.77
	dinner	10	21.28	24	21.24	1	0.44–2.30
18th June	breakfast	10	21.28	35	30.97	0.6	0.27–1.35
	lunch	32	68.09	83	73.45	0.77	0.37–1.62
	dinner	14	29.79	23	20.35	1.66	0.77–3.6
19th June	breakfast	10	21.28	29	25.66	0.78	0.35–1.77
	lunch	46	97.87	73	64.60	25.21	3.35–189.69
	dinner	12	25.53	17	15.04	1.94	0.84–4.46
	Red bean cake (the pastry at lunch)	45	95.74	2	1.77	1248.75	170.64–9138.33

^15 cases were excluded from the case-control analysis due to non-participation in exposure assessments.^

^2Control count exceeds 1:2 ratio due to retention of all eligible controls within matched strata for 19 cases.^

^3OR: odds ratio.^

^4CI: confidence interval.^

#### 3.5.4 Laboratory test results

**Figure 3:**
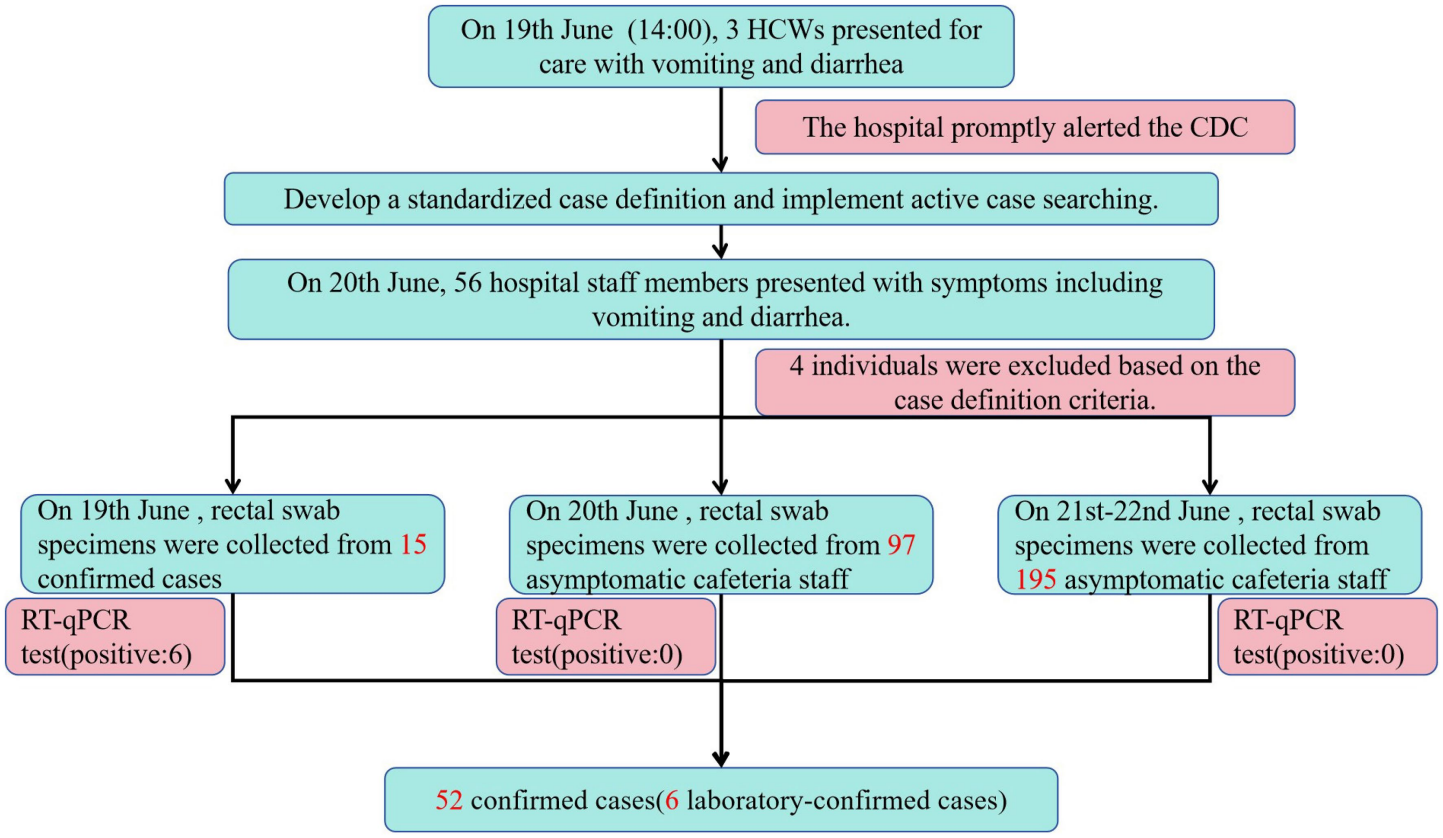
Outbreak response flowchart of norovirus GII in a Zhejiang hospital, June 2024.

On 19th June, rectal swabs were collected from 15 individuals (14 HCWs and 1 cafeteria manager) presenting for clinical evaluation at the hospital’s outpatient department. Of 22 cases with active symptoms on 19th June, 7 cases did not seek medical care due to mild symptoms. Consequently, specimens were obtained only from clinically assessed cases and 10 environmental swabs collected from cafeteria food contact surfaces prior to disinfection on 19th June. Norovirus GII RNA was detected in rectal swabs from 5 HCWs, the cafeteria manager, and the cafeteria-sourced “red bean cake” consumed at noon on 19th June (Cycle threshold values in Table 6). All 10 swabs from post-processing preparation surfaces tested negative for norovirus. This suggests contamination likely occurred immediately before service. To rule out asymptomatic carriers as potential transmission sources, 97 rectal swabs were collected from cafeteria staff on 20th June. An additional 195 swabs were obtained during 21st–22nd June to cover all 103 cafeteria employees (including repeated tests for high-risk roles). All 292 samples tested negative for norovirus, excluding cafeteria staff as reservoir of ongoing transmission. No asymptomatic norovirus-positive individuals were identified during screening. Bacterial cultures of all 52 clinical specimens uniformly yielded no growth for *Bacillus cereus*, Salmonella spp., Shigella spp., *Staphylococcus aureus*, *Vibrio parahaemolyticus*, or Campylobacter spp. No bacterial pathogens were detected in any clinical specimens from norovirus-positive cases, confirming the absence of viral-bacterial co-infections. The outbreak response protocol is summarized in the epidemic control flowchart (Figure 3).

**Table 6:** Norovirus RT-qPCR positive samples and Ct values.

Positive samples	Ct^1^
case 1	30
case 2	31
case 3	31
case 4	34
case 5	38
case 6	33
red bean cake	38

^1Ct: Cycle threshold values.^

## 4 Discussion

This investigation definitively identified the cafeteria-sourced “red bean cake” as the primary transmission vehicle for the nosocomial norovirus GII outbreak. Consumption of red bean cake conferred an high risk magnitude (OR = 1248.75, 95% CI: 170.64–9138.33), This extraordinary effect size was corroborated by RT-qPCR detection of norovirus GII RNA in food samples. These findings exposed critical vulnerabilities in hospital food safety protocols and revealed salient epidemiological features of norovirus transmission in healthcare settings. Notably, no secondary transmission to inpatients occurred in affected departments, indicating effective infection control practices (Immediate self-isolation of symptomatic staff, strict hand hygiene adherence and enhanced environmental disinfection in clinical areas using 1,000 mg/L chlorine-based agents) among HCWs. This epidemiological pattern further suggests potential deficiencies in cafeteria hygiene standards.

Norovirus GII RNA was identified in rectal swabs from HCWs and cafeteria staff, as well as in cafeteria food samples. Triangulation of epidemiological patterns, clinical presentations, and laboratory findings confirmed this outbreak as norovirus-associated. The outbreak originated from cafeteria-sourced food, with transmission occurring via foodborne routes to HCWs.

The nosocomial norovirus outbreak (*n* = 52) was successfully contained within 96 h of case identification, with rapid source attribution achieved through multimodal interventions including case isolation, environmental disinfection, and staff screening. A 2020 UK-based systematic review of 72 hospital-associated norovirus outbreaks reported a median containment duration of 18 days (IQR: 12–24 days) (17). In contrast, a Dutch multicenter cohort study (2009) of 37 outbreaks across 42 healthcare facilities demonstrated significantly shorter containment periods (median 12 days, 95% CI: 9–15 days) (18). The observed disparity in containment efficacy may reflect distinct risk profiles of exposed populations—HCWs versus community-acquired cases. HCWs typically exhibit higher adherence to infection control protocols and enhanced pathogen transmission awareness. Consequently, outbreaks involving predominantly HCWs demonstrate accelerated resolution due to higher adherence to infection control protocols, consistent with studies reporting reduced transmission in settings with optimized interventions (17). Notably, norovirus cluster outbreaks exhibit distinct seasonality, with winter months (December–February) accounting for 72% of healthcare-associated outbreaks in temperate regions (19). However, this outbreak occurred during summer (June–August) when ambient temperatures exceeded 25°C, a condition shown to reduce norovirus environmental stability compared to winter conditions (20).

The outbreak stemmed from foodborne transmission, with the cafeteria-served “red bean cake” epidemiologically implicated as the infection source. Affected cases exhibited cardinal symptoms of norovirus gastroenteritis. RT-qPCR confirmed norovirus GII nucleic acid in 6 of 15 clinically suspected cases (40.0%). Symptom onsets clustered within a 36-h window, strongly supporting a point-source exposure. All six laboratory-confirmed norovirus cases (19th June) reported consumption of the cafeteria-served “red bean cake” during the 12:00–13:00 h exposure window, with unequivocal recall of food ingestion timing. The case-control study demonstrated a 25.21-fold increased risk (OR = 25.21, 95% CI: 3.35–189.69) for 19th June cafeteria lunch attendance, aligning with the 12–48-h incubation period typical of norovirus gastroenteritis. Symptom onsets clustered within 36 h post-exposure, while negative bacterial cultures confirmed norovirus as the definitive etiological agent. Stratified multivariable analysis of the implicated “red bean cake” revealed an exceptionally elevated risk (OR = 1248.75, 95% CI: 170.64–9138.33), surpassing the OR threshold of 50 that demarcates high-risk foodborne events (21). A key methodological strength was the prompt implementation of a 1:2 matched case–control design (≤24 h post-index case confirmation), significantly reducing recall bias through standardized exposure questionnaires validated in prior norovirus outbreaks.

Norovirus RNA was detected through RT-qPCR in 6 of 15 clinical specimens (40.0%) collected from early-presenting cases. Although RT-qPCR represents the gold standard for norovirus detection owing to its high sensitivity (typically >90%) and specificity (>95%) under optimized conditions (22–24), several factors could account for the 60% negative rate in this subset. Viral shedding peaks during the acute phase (24–48 h post-symptom onset) before declining rapidly (25). Consequently, samples collected >72 h post-onset might fall below detection limits. Inadequate fecal sample collection or interruptions in cold-chain transport could degrade RNA. Despite high overall accuracy, primer/probe mismatches against emerging GII variants may reduce sensitivity (26). Fecal samples harbor PCR inhibitors that might evade purification (27). Critically, norovirus GII RNA was detected in the epidemiologically implicated “red bean cake,” while all laboratory-confirmed norovirus cases reported consumption of this item. The convergence of epidemiological, clinical, and laboratory evidence including the food sample detection mitigates concerns regarding potential false negatives in clinical testing.

These findings revealed critical vulnerabilities in hospital food safety protocols, particularly concerning non-thermally processed or post-thermally contaminated ready-to-eat foods. The epidemiological and laboratory evidence conclusively implicates the cafeteria-produced “red bean cake” as the outbreak vehicle. Although the precise contamination point could not be determined retrospectively, several plausible pathways correspond with norovirus transmission dynamics and the outbreak’s point-source pattern. Field investigation of the ‘red bean cake’ preparation process identified multiple potential contamination pathways (Figure 2). While retrospective analysis could not definitively establish the contamination source, epidemiological and operational evidence points strongly to bare-hand contact during cutting and portioning as the critical failure point. This step occurred after baking processing, which would have inactivated any norovirus present in raw ingredients. Given norovirus’s low infectious dose and environmental stability, transient viral shedding from an asymptomatic and pre-symptomatic handler during this step could readily contaminate the product. Although all cafeteria staff tested negative post-outbreak, this does not preclude pre-symptomatic shedding during preparation (likely 18–19 June, based on incubation periods). Alternative pathways contaminated raw ingredients or water are less probable, as thermal processing should have eliminated viral load, and no irregularities in supplier documentation or water systems were observed. Notably, four cafeteria management staff (non-food-handling personnel) developed symptoms on 19th June. Although not directly involved in food handling, their presence in preparation or storage areas suggests potential environmental shedding or indirect contamination through fomites. Given norovirus’s environmental stability, contaminated raw ingredients might have introduced the virus before thermal processing. Although the cafeteria reported using commercially sourced ingredients, supplier-level traceability and pre-delivery testing data were unavailable during this investigation. Additionally, water used in preparation was not tested before the outbreak.

This study’s limitations include the absence of genomic sequencing on clinical and food specimens. This omission precluded confirmation of genetic linkage between human-derived and foodborne viral strains; consequently, transmission chain reconstruction was impeded. Second, our symptom-based case definition excluded asymptomatic individuals. While screening of 292 asymptomatic cafeteria staff (20st–22nd June) yielded no norovirus positives, it remains possible that asymptomatic shedders among the wider staff population were missed. This limitation could lead to underascertainment of outbreak magnitude and potential underestimation of transmission dynamics. Third, post-disinfection environmental sampling likely underestimated pre-intervention contamination levels, as residual disinfectants reduce RT-qPCR sensitivity (28). Laboratory sampling was limited to cases seeking clinical care, potentially underestimating norovirus prevalence among mild or non-presenting cases.

## 5 Conclusion

These findings highlight the necessity for stringent oversight of healthcare facility food services, specifically rigorous monitoring of non-thermally processed foods. Mandatory pre-employment training, validated health certifications, and daily health surveillance for cafeteria staff are essential to ensure compliance with hygiene protocols. Cafeteria personnel must not engage in food handling without valid health certifications or while exhibiting symptoms of illness. Water safety within the cafeteria’s culinary and potable water systems necessitates rigorous monitoring to prevent microbial contamination. Hospital food service areas represent high-risk environments for enteric infection outbreak clusters, given frequent pathogen exposure. Specifically, hospitals must prohibit bare-hand contact with ready-to-eat foods, mandating gloves/utensils and Audit post-thermal processing steps for contamination risks. Routine audits of disinfection protocols by the infection control department are critical to ensure compliance with hygiene standards. These measures provide evidence-based strategies for preventing and managing nosocomial enteric infections.

## Data Availability

The original contributions presented in the study are included in the article/Supplementary material, further inquiries can be directed to the corresponding authors.
