# Supplementary material for: Investigation of a norovirus outbreak among hospital staff in Zhejiang, China: tracing the source to contaminated “red bean cake”
Source: Front Public Health. 2025 Jul 24;13:1631091. doi: 10.3389/fpubh.2025.1631091 (PMC12328458; doi:10.3389/fpubh.2025.1631091)
Supplement: Supplementary file 1 [file Data_Sheet_1.pdf]

SUPPLEMENTAL MATERIAL

Investigation of source of a norovirus outbreak among healthcare workers in Zhejiang,China

Supplementary Table 1. Formulation protocol for the dual reaction system of norovirus(QuantiTect Multiplex RT－PCR Kits)

Component	Volume(μl)	Final concentration(nM)
RNase-free water	3	
Cog 1F（10μM）	1	400
Cog 1R（10μM）	1	400
Ring 1A（10μM）	0.5	200
Cog 2F（10μM）	1	400
Cog 2R（10μM）	1	400
Ring 2（10μM）	0.5	200
RNase-free water	4.25	
QuantiTect Multiplex RT Mix	0.25	
2×QuantiTect Multiplex RT-PCR Master Mix	12.5	1x

Supplementary Table 2. Primers and probes for multiplex fluorescent quantitative RT-PCR amplification of norovirus

Genotype	Primers/probes	Sequence (5’-3’)
GI	Cog 1F	CGY TGG ATG CGN TTY CAT
	Cog 1R	CTT AGA CGC CAT CAT CAT TYA C
	Ring 1A	FAM-AGA TYG CGA TCY CCT GTC CA-BHQ1
GII	Cog 2F	CARGAR BCN ATG TTY AGR TGG ATG AG
	Cog 2R	TCG ACG CCA TCT TCA TTC ACA
	Ring 2	CY5-TGG GAG GGC GAT CGC AAT CT-BHQ2

Supplementary Table 3 Clinical manifestations of 52 cases in a norovirus enteritis outbreak among healthcare workers, Zhejiang, 2024

Clinical manifestations	Number of cases	Rate（%）
Diarrhea	50	96.15
Vomiting	34	65.38
Abdominal pain	18	34.62
Fever	13	25
Nausea	3	5.77

Supplementary Table 4 Case characteristics in a norovirus enteritis outbreak among healthcare workers, Zhejiang, 2024

Characteristics	Groups	Number of cases	Attack rate (%)	χ^2	*P*
Gender	Male	11	1.77	0.77	0.38
	Female	41	2.37		
Occupation	healthcare workers(HCWs)	48	2.30	0.43	0.51
	Canteen staff	4	0.76		
Building	Administration building	8	2.04	0.06	0.80
	Medical building	44	2.30		
Floor	Low	34	2.46	2.21	0.33
	Middle	7	1.33		

	High	9	2.22	

Supplementary Table 5 Exposure analysis of meal times and food items in a norovirus outbreak among healthcare workers, Zhejiang, 2024

date	exposure factors	cases（n=47[1]）		control（n=113[2]）		OR[3]	95%CI[4]
		exposed population	exposure rate（%）	number of exposed people	Exposure rate（%）		
June 17th	breakfast	9	19.15	35	30.97	0.53	0.23~1.21
	lunch	24	51.06	83	73.45	0.38	0.19~0.77
	dinner	10	21.28	24	21.24	1	0.44~2.30
June 18th	breakfast	10	21.28	35	30.97	0.6	0.27~1.35
	lunch	32	68.09	83	73.45	0.77	0.37~1.62
	dinner	14	29.79	23	20.35	1.66	0.77~3.6
June 19th	breakfast	10	21.28	29	25.66	0.78	0.35~1.77
	lunch	46	97.87	73	64.60	25.21	3.35~189.69
	dinner	12	25.53	17	15.04	1.94	0.84~4.46
	Red bean cake (the pastry	45	95.74	2	1.77	1248.75	170.64~9138.33

1 5 cases were excluded from the case-control analysis due to non-participation in exposure assessments.

2 Control count exceeds 1:2 ratio due to retention of all eligible controls within matched strata for 19 cases

3 OR:odds ratio

4 CI::confidence interval

at lunch

Supplementary Table 6 Norovirus RT-qPCR positive samples and Ct values

positive samples	Ct[5]
case 1	30
case 2	31
case 3	31
case 4	34
case 5	38
case 6	33
red bean cake	38

[5] Ct:Cycle threshold values
